# Remission in chronic rhinosinusitis: Defining the construct, and considerations for its application in practice

**DOI:** 10.1016/j.bjorl.2026.101933

**Published:** 2026-10-23

**Authors:** Ahmad R. Sedaghat, Wilma Terezinha Anselmo-Lima

**Affiliations:** a University of Cincinnati College of Medicine, Department of Otolaryngology–Head and Neck Surgery, Cincinnati, OH, United States; b Universidade de São Paulo (USP), Faculdade de Medicina de Ribeirão Preto (FMRP), Department of Ophthalmology, Otorhinolaryngology and Head and Neck Surgery, Division of Otorhinolaryngology, Ribeirão Preto, SP, Brazil

Recently, the concept of remission has been introduced into the vocabulary for chronic rhinosinusitis with nasal polyps (CRSwNP) and proposed as a desirable goal of treatment, primarily in the context of biologics.^1^ The addition of remission to the lexicon of chronic rhinosinusitis outcomes is conceptually important but requires a thoughtful approach. New terminology should provide added value to a field’s dialogue by having a meaning that is distinct from existing terminology. New treatment goals should provide a unique, meaningful benefit for the patient and should not be implicitly linked to a specific therapeutic class. Without these qualifications, the application of remission to CRSwNP may seem arbitrary, redundant and influenced by industry bias.

First, it is important to highlight that years of treatment advances and scientific study have established that CRSwNP is no longer viewed as merely a recurrent surgical disease. It is increasingly recognized as a chronic inflammatory disorder, often associated with type 2 inflammation and manifestations in other compartments of the airways. Within this new paradigm, established outcomes and goals of treatment such as control, response, recurrence, progression, and disease modification, as well as new ones such as remission may represent related terms but must represent non-equivalent states.

To date, however, the application of the term remission to CRSwNP has in many cases appeared to redundantly reflect controlled disease, rather than a novel, unique disease state. Remission cannot simply be “control under another name.” Over the last two decades, the terminology of remission has been introduced for other chronic inflammatory diseases and one common, explicitly stated requirement has been that disease remission must have a definition that is distinct from other outcomes and treatment goals, such as disease control. For example, consensus studies focusing on remission in asthma have reached full consensus that the introduction of remission as a new terminology must include a clear distinction between it and the construct of disease control.

We believe that the construct of ***remission should reflect a cure-like disease state with no or very minimal disease activity that is distinguished from cure by only the possibility of disease recurrence***. In other words, a patient in remission does not have obvious manifestations of their disease. This definition is consistent with how remission has been defined for many other chronic inflammatory diseases over the last two decades.^2^ This definition also establishes a clear delineation between the constructs of remission and control, with ***control reflecting a disease state in which disease activity and manifestations are acceptable but not necessarily non-existent***. In fact, many studies have now demonstrated that acceptable disease manifestations for chronic rhinosinusitis are clearly inclusive of obvious, detectable disease activity.^3^ Disease control and remission are therefore distinguished by the permissibility of disease activity and manifestations by the former but not by the latter.

However, even the proposed definition for the construct still leaves important questions unresolved in the application of remission as an outcome and goal of treatment for CRSwNP. If remission reflects a cure-like state with no detectable disease activity — for example, characterized by no symptoms or visible nasal polyps, what additional benefits does it provide for the patient that is above and beyond a state of acceptable disease activity that is reflected by disease control? Does suppression of CRSwNP disease activity to achieve remission provide direct benefit to the patient? For example, does suppression of controlled CRSwNP symptoms to achieve no detectable symptoms provide added quality of life (QOL) benefit for patients? Does further treatment of endoscopically controlled CRSwNP to eliminate all nasal polyps improve QOL for patients? Compared to controlled CRSwNP, does remission provide a lower risk of recurrence, slower disease progression, less future need for surgery or systemic corticosteroids, and a better long-term prognosis? At present, we are unaware of any robust scientific evidence that would indicate suppression of CRSwNP disease activity to achieve remission will provide clear benefit to patients above and beyond achieving controlled CRSwNP. As such and in the absence of evidence, should we be exposing patients with controlled CRSwNP to more treatments to further suppress their disease?

Another issue that deserves careful consideration is the relationship between remission and treatment burden. Remission while receiving treatment and remission without treatment are not equivalent states. Whether remission maintained on treatment is a worthwhile goal will depend on the benefit imparted on the patient. For example, if remission provides significant quality of life benefit to patients compared to controlled CRSwNP, then achieving of remission on treatment may be a desired goal. On the other hand, if the benefit of remission is the achievement of a disease state that is en route to cure but does not necessarily provide other noticeable benefit to the patient, then remission off treatment may be most pertinent. However, these considerations further highlight the necessity for identifying what is the clear benefit and utility for remission in CRSwNP.

The treatment class most touted as the means to achieve CRSwNP disease remission has been biologics. However, the definition for the construct of remission is consistent with an essential principle: remission should be defined by the clinical state achieved by the patient, not by the treatment used to achieve it. Appropriately indicated endoscopic sinus surgery followed by adequate topical treatment, may also lead to prolonged periods without clinically relevant symptoms, visible polyps, systemic corticosteroid use, or the need for further intervention. In some patients, surgery may be the main pathway to remission; in others, a biologic may be required to induce or maintain it; and some patients may need a combined strategy. Remission should not be considered an exclusive privilege of biologic therapy.

Given the recent promotion of biologics as the primary treatment tool for achieving CRSwNP remission in the absence of robust evidence to support the utility of pursuing remission in patients with already controlled disease, the exclusion of industry influence is an important consideration. Although collaboration between academia and industry has contributed substantially to the development of new therapies, the definition of remission in CRSwNP should remain grounded in robust, clinically meaningful, and patient-centered scientific criteria. As different stakeholders may have distinct priorities, the outcomes used to define disease control or remission should be clearly established, validated, and applied transparently. In this process, scientific rigor, academic independence, and meaningful clinical benefit for patients should remain the primary considerations.

Remission in CRSwNP is a promising concept, but it remains a work in progress. We believe that it could be a useful construct as a cure-like state of absent disease activity or minimally active disease with absent clinical manifestations, sustained over time, measurable using well-defined evidence-based criteria, and associated with a meaningful prognostic benefit. It should also remain independent of the therapeutic strategy used — surgery, biologic therapy, topical treatment, or a combination of these approaches. However, this terminology must be used rigorously. The challenge is not merely to adopt a new word, but to demonstrate that it provides added value by reflecting a distinct and novel construct that is genuinely meaningful to the patient, improves the dialogue in our field, and is free of other external influences.

## ORCID ID

Ahmad R. Sedaghat: 0000-0001-6331-2325

## Funding

None.

## Data availability

The authors declare that all data are available in repository.

## Declaration of competing interest

The authors declare no conflicts of interest.
